# Exploring the vegetation of the coastal road in Puerto Cisnes, southern Chile: a vascular plant inventory

**DOI:** 10.3897/BDJ.11.e107217

**Published:** 2023-08-21

**Authors:** Jimmy Pincheira-Ulbrich

**Affiliations:** 1 Departamento de Ciencias Ambientales, Facultad de Recursos Naturales, Universidad Católica de Temuco. Rudecindo Ortega 02950, Temuco, Chile Departamento de Ciencias Ambientales, Facultad de Recursos Naturales, Universidad Católica de Temuco. Rudecindo Ortega 02950 Temuco Chile; 2 Núcleo de Estudios Ambientales, Universidad Católica de Temuco, Temuco, Chile Núcleo de Estudios Ambientales, Universidad Católica de Temuco Temuco Chile; 3 Laboratorio de Planificación Territorial, Universidad Católica de Temuco, Temuco, Chile Laboratorio de Planificación Territorial, Universidad Católica de Temuco Temuco Chile

**Keywords:** invasive species, filmy ferns, vascular plants, rupicolous plants, fjords

## Abstract

**Background:**

In areas of low disturbance, such as the Aysén Region of Chile, the presence of roads can inadvertently facilitate the spread of invasive species. To address this issue, it is imperative to maintain up-to-date biological inventories, as they serve as a primary source of information for the conservation of species and ecosystems. However, the maintenance of systematic inventories of vascular plants in Chile is virtually non-existent, especially outside protected wilderness areas. The data we have come from an inventory of vascular plant species along a stretch of coastal road in Puerto Cisnes (Aysén Region), characterised by a cut slope in the rock. The site is located between mountain ranges, in a region known for its protected wilderness areas and low levels of anthropogenic alteration. The study adopted an observational sampling design, using the road as a transect. For each species identified, the growth substrate, habit and dispersal mode were recorded. A total of 70 species (36 herbs, 23 shrubs and 11 trees) belonging to 42 families were found. The most represented families were Hymenophyllaceae (nine species) and Myrtaceae (four species). We recorded nine introduced species belonging to seven botanical families (*Cirsiumvulgare* (Savi) Ten., *Crocosmiacrocosmiiflora* (Lemoine ex Burb. & Dean) N.E.Br., *Cytisusscoparius* (L.) Link, *Digitalispurpurea* L., *Lotuspedunculatus* Cav., *Plantagolanceolata* L., *Polygonumcampanulatum* Hook. f., *Prunellavulgaris* L., *Rubusconstrictus* Lefèvre & P.J.Müll). Of these nine species, seven are invasive, while the remaining two species have not been assessed for invasive potential (i.e. *Crocosmiacrocosmiiflora* and *Polygonumcampanulatum*). In particular, *Crocosmiacrocosmiiflora* and *Rubusconstrictus* are new regional records. The majority of species were found growing on the ground (44 species), while a significant proportion were found exclusively on rocky slopes (17 species). According to their seed dispersal mechanism, the most common syndromes were anemochory (32 species) and ornithochory (20 species). Other mechanisms such as mammalochory, ballochory or myrmecochory were less common (less than four species).

**New information:**

This study provides valuable data on the vascular flora of Puerto Cisnes, Chile, a modest human settlement in a minimally altered landscape. The region, dominated by native forests and a burgeoning salmon farming industry, has few inventories, so the database presented here adds significantly to local botanical knowledge. The main novelty of this research is that it is the first inventory carried out on a road in a slightly altered area surrounded by protected wilderness areas (such as Magdalena Island National Park and Queulat National Park). The study systematically categorises species according to substrate, habitat and dispersal mode, dimensions that are rarely combined in a single database.

The inventory identifies 70 species (36 herbs, 23 shrubs and 11 trees) in 42 families. The most represented families were Hymenophyllaceae (with nine species) and Myrtaceae (with four species). Additionally, we recorded, two introduced species (*Crocosmiacrocosmiiflora* and *Rubusconstrictus*) at least 100 km south of their known distribution.

## Introduction

Inventories are indispensable for understanding the spatial and temporal distribution of species. Such baseline information can serve multiple purposes, such as the generation of species distribution models (Weigelt et al. 2019), ecosystem restoration (Rai 2022) and the management and control of invasive exotic species (Fuentes et al. 2010). In Chile, as in many other Latin American countries, species monitoring is virtually non-existent (Möller and Muñóz-Pedreros 2014, Fuentes et al. 2010, Moussy et al. 2021), leaving little information to track the movement of species or their populations within a region. Certain groups, such as invasive plants, can significantly impact ecosystems, nutrient cycling, water production and fire regimes (Weidlich et al. 2020). These species colonise open areas, such as roadsides or railway lines (Deeley and Petrovskaya 2022), but systematic monitoring of these types of sites is lacking (Weigelt et al. 2019).

Inventories conducted on roads facilitate the study of species movements and their dispersal to other sites, enabling the prevention and management of potential biological invasions (e.g. Pauchard and Alaback 2004, Fuentes et al. 2013, Deeley and Petrovskaya 2022). Inventories are, therefore, needed to identify which new species are establishing in a given area and to act as an early warning system to prevent potential impacts. The identification and control of invasive species can contribute to the 14^th^ and 15^th^ goals of the Sustainable Development Goals (United Nations 2015) and, in particular, to the post-2020 global biodiversity framework (CBD 2021).

The Aysén Region of Chile has a limited number of inventories (e.g. Tomé et al. (2007), Teillier and Marticorena (2002), Quintanilla et al. (2008), Rodríguez et al. (2008), Promis et al. (2013), Sánchez-Jardón et al. (2013), Ramírez et al. (2014)); therefore, the database presented in this work contributes to the local understanding of the flora. The main novelty of this study is that it is the first inventory carried out on a road in a slightly modified area surrounded by protected wilderness areas (i.e. Magdalena Island National Park and Queulat National Park). The study includes the systematic categorisation of species based on substrate, habitat and dispersal mode, aspects rarely reported in a single database (but see Pincheira-Ulbrich et al. (2021)).

The inventory presents a total of 70 species (36 herbs [Fig. 5], 23 shrubs [Fig. 6] and 11 trees [Fig. 7]) belonging to 42 families (Table 1, Suppl. material 1). We recorded nine introduced species belonging to seven botanical families. Of these nine species, seven are invasive (*Cirsiumvulgare* (Savi) Ten., *Cytisusscoparius* (L.) Link, *Digitalispurpurea* L., *Lotuspedunculatus* Cav., *Plantagolanceolata* L., *Prunellavulgaris* L., *Rubusconstrictus* P.J. Müll. & Lefèvre), while the remaining two species (i.e. *Crocosmiacrocosmiiflora* and *Polygonumcampanulatum*) have not been assessed for invasive potential (Fuentes et al. 2013, Fuentes et al. 2020). The most represented families were Hymenophyllaceae (nine species, Fig. 4) and Myrtaceae (four species, e.g. Fig. 7a, e). Two new records of introduced species (*Crocosmiacrocosmiiflora* and *Rubusconstrictus*, Fig. 3) were recorded at least 100 km south of their known distribution (Fuentes et al. 2013, Rodriguez et al. 2018, Fuentes et al. 2020).

## Project description

### Study area description

**Figure 1.**
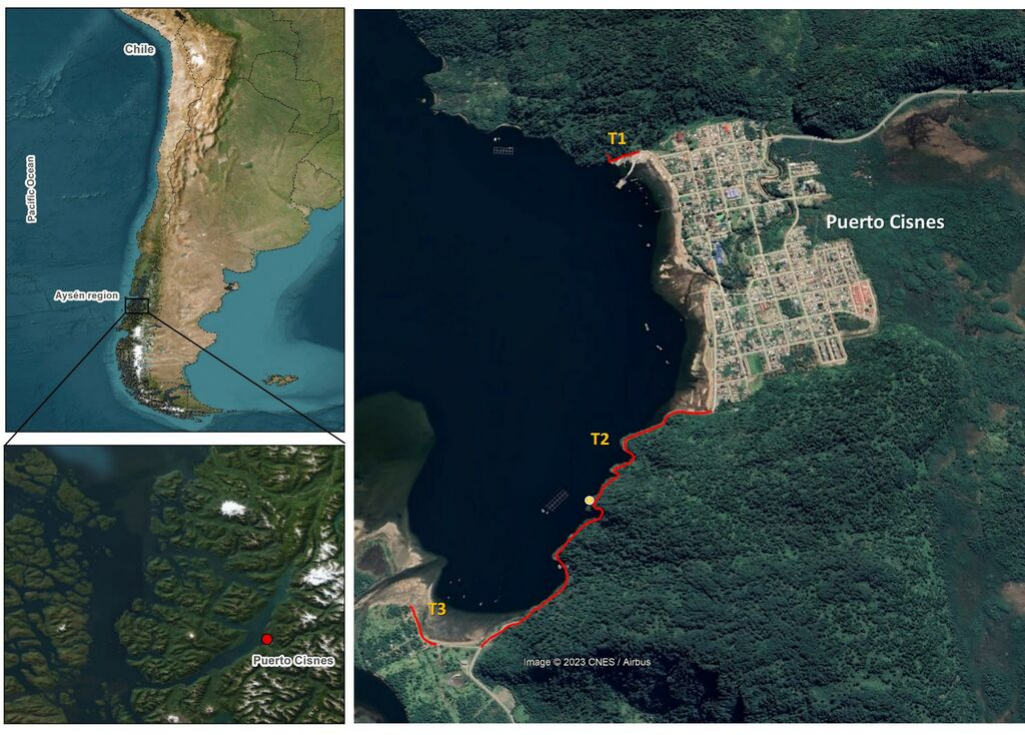
Study area in Puerto Cisnes. T1: transect 1, corresponding to a small transect in the north. T2: transect 2, corresponding to most of the road. Yellow circle, corresponding isolated rock in the middle of transect 2. T3: transect 3, corresponding to the beach in the south.

Puerto Cisnes is a small coastal town in the Aysén Region of Chile (44°43'46.33"S, 72°40'51.85"W). It is located in a small bay of the Puyuhuapi Channel, adjacent to the mouth of the Cisnes River (Fig. 1), opposite the Magdalena Island National Park and a few kilometres from the Queulat National Park. The landscape is diverse and includes native forests, channels and mountain ranges. The town has a population of about 7,000 and the salmon industry is the main economic activity, with tourism a secondary activity.

### Design description

Data collection took place between 24 and 26 February 2017. Sampling followed an observational protocol using the road as a transect with a continuous walk-through approach being employed (Brower et al. 1997). An inventory of species occurrences was conducted along a transect of approximately 2.45 km, covering both sides of the road and the rock face forming the fjord escarpment. In the near-vertical cut areas adjacent to the road, species growing within the first few metres, easily accessible from the road, were recorded. Whilst safety constraints prevented direct sampling from higher areas, an attempt was made to identify all species visible from the base of the cut. Each species was recorded at the time of first detection, regardless of subsequent occurrences within the transect. The primary aim of this strategy was to capture the broadest possible diversity of species within the constraints of the study area (Diekmann et al. 2007). The width of the transect was dictated by the physical constraints of the site, namely the road and the adjacent rock face (Diekmann et al. 2007, Speak et al. 2018). On the beach, the average transect width was 10 m.

The data were organised according to four sampling locations: Transect 1, corresponding to a small transect in the north; Transect 2, corresponding to most of the road; Isolated Rock, corresponding to a point in the middle of Transect 2; and Transect 3, corresponding to the beach in the south (Fig. 2). The urban area was excluded from the study. After data collection, the information was formatted according to the Darwin Core Standard for Biodiversity Data (https://dwc.tdwg.org/). The refinement of this criterion by Groom et al. (2019) enhances its suitability for the study and management of invasive species by providing a more detailed representation of the native status, establishment level and site occupancy means of the organism.

## Sampling methods

### Sampling description

Field notes, photographs and some difficult-to-identify specimens taken along the transects were examined in the laboratory. Three types of data were described: (i) taxonomic identity, according to Marticorena and Rodríguez (Marticorena and Rodríguez 1995, Marticorena and Rodríguez 2001, Marticorena and Rodríguez 2003, Marticorena and Rodríguez 2005, Marticorena and Rodríguez 2011), (ii) microhabitat substrate (soil, rock escarpment, tree) as observed in the field, (iii) growth form (climber, epiphyte, liana, terricolous), according to Rodriguez et al. (2018), (vi) habit (herb, shrub, subshrub and tree), according to Rodriguez et al. (2018), (vi) dispersal syndrome (anemochorous, ornithochorous, mammalochory, ballochory, myrmecochory), according to Armesto and Rozzi (1989), Wilson et al. (1996), Salvande et al. (2011) and (vi) geographic origin (native, endemic, introduced) according to Rodriguez et al. (2018) and Fuentes et al. (2020). Taxonomic nomenclature followed Rodriguez et al. (2018) and the International Plant Names Index (IPNI 2022).

## Geographic coverage

### Description

Locality of Puerto Cisnes in Chile, situated in a small bay of the Puyuhuapi Channel, next to the mouth of the Cisnes River.

### Coordinates

-44.7454° and -44.7242° Latitude; -72.6989° and -72.6877° Longitude.

## Usage licence

### Usage licence

Creative Commons Public Domain Waiver (CC-Zero)

## Data resources

### Data package title

Vascular plants along a coastal road in Puerto Cisnes, Aysén Region, Chile.

### Number of data sets

1

### Data set 1.

#### Data set name

Vascular plants along a coastal road in Puerto Cisnes, Aysén Region, Chile.

#### Description

The dataset lists 70 vascular plant species found in three transects made along a rural road in Puerto Cisnes, Chile (Suppl. material 1).

**Data set 1.** 

Column label	Column description
occurrenceID	A unique identifier for each occurrence.
scientificName	The scientific name of taxon.
scientificNameAuthorship	The authorship information for the scientific name.
kingdom	The full scientific name of the kingdom in which the taxon is classified.
class	The full scientific name of the class in which the taxon is classified.
order	The full scientific name of the order in which the taxon is classified.
family	The full scientific name of the family in which the taxon is classified.
habitat	Habitat type where species was observed (i.e. Road in an evergreen forest, Beach path).
locationRemarks	Comments or notes about the location (i.e. Growing on rock, growth in the soil, tree bark or a combination of these).
country	The name of the country where the organism was found.
municipality	Village around which sampling was carried out.
stateProvince	The administrative region where sampling took place.
eventRemarks	Name of the street where the transect was located.
locality	The specific mention of the sampling unit in which the organism was found (Transects 1, 2, 3 or isolated rock).
samplingProtocol	Name of the protocol used during sampling.
decimalLatitude	The latitude of the centre of each locality.
decimalLongitude	The longitude of the centre of each locality.
dynamicProperties	A list of additional measurements for the record. Seed dispersal syndrome, Growth form, Habit.
establishmentMeans	Statement about whether a organism has been introduced to a given place and time through the direct or indirect activity of modern humans (i.e. native, introduced).
degreeOfEstablishment	The degree to which a organism survives, reproduces and expands its range at the given place and time (i.e. native, invasive, casual, established).
geodeticDatum	The geographic coordinates given in decimal latitude and decimal longitude are based on a specific ellipsoid, geodetic datum or spatial reference system (SRS) (i.e. WGS84).
coordinateUncertaintyInMetres	Measurement uncertainty in metres.
eventDate	The date when the organism was registered.
recordedBy	Name of the observer.
recordedByID	Unique identifier of the species identifier in ORCID.

## Supplementary Material

69D9AB1F-5EA0-5396-A4E6-4DCFC16F96D0 10.3897/BDJ.11.e107217.suppl1 8275626 Supplementary material 1 Vascular plants along a coastal road in Puerto Cisnes, Aysén Region, Chile Data type occurrences Brief description Vascular plants along a coastal road in Puerto Cisnes, Aysén Region, Chile. File: oo_881785.tsv https://binary.pensoft.net/file/881785 Jimmy Pincheira-Ulbrich

## Figures and Tables

**Figure 2a.**
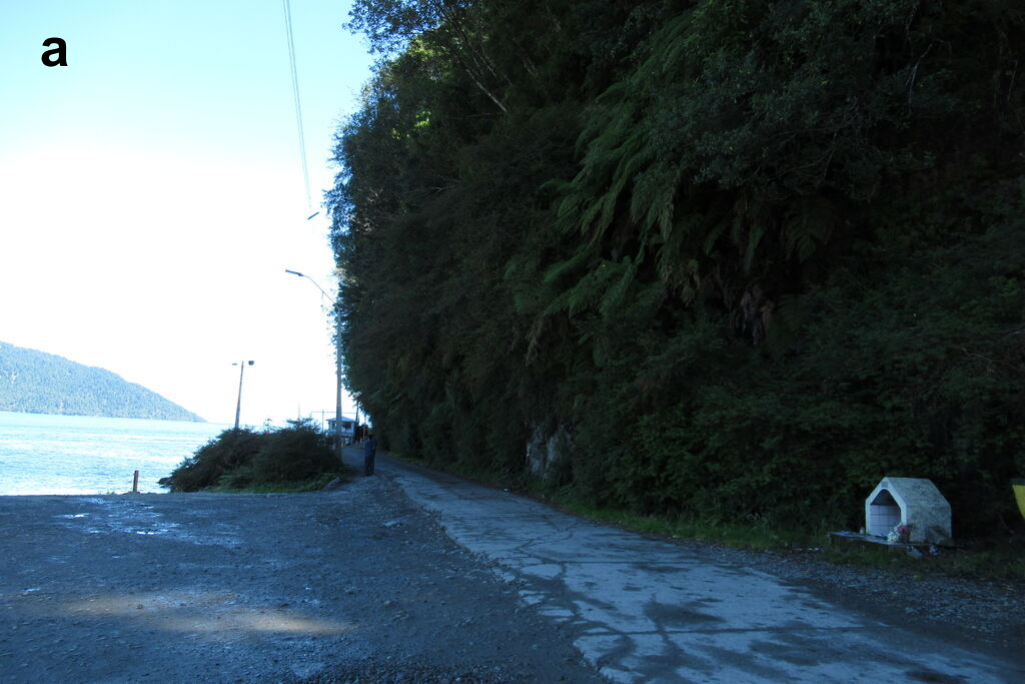
Transect 1;

**Figure 2b.**
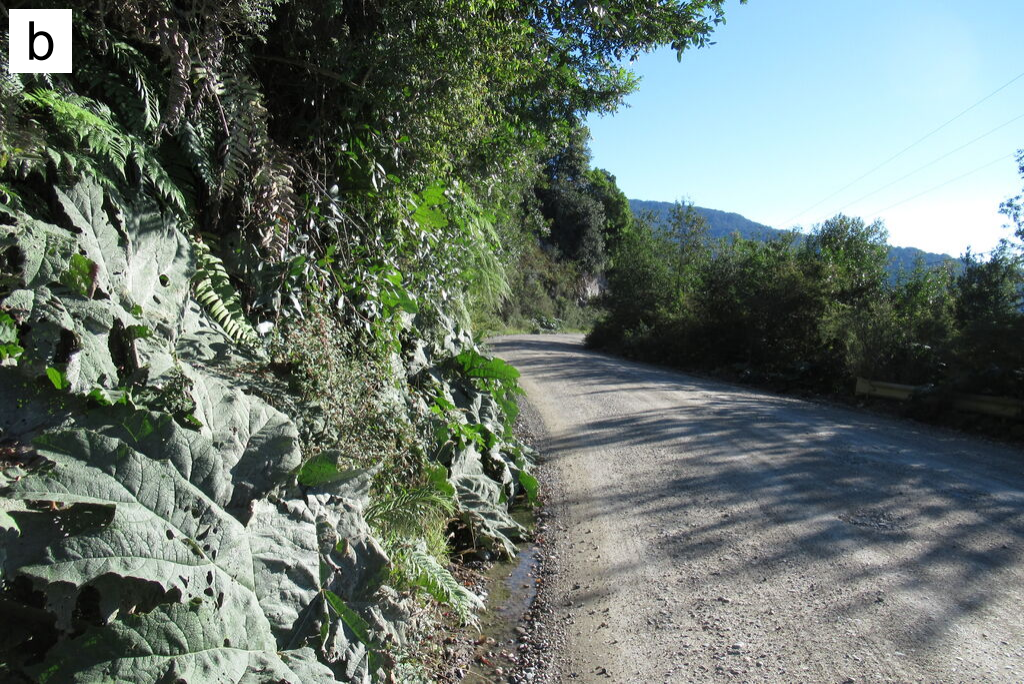
Transect 2;

**Figure 2c.**
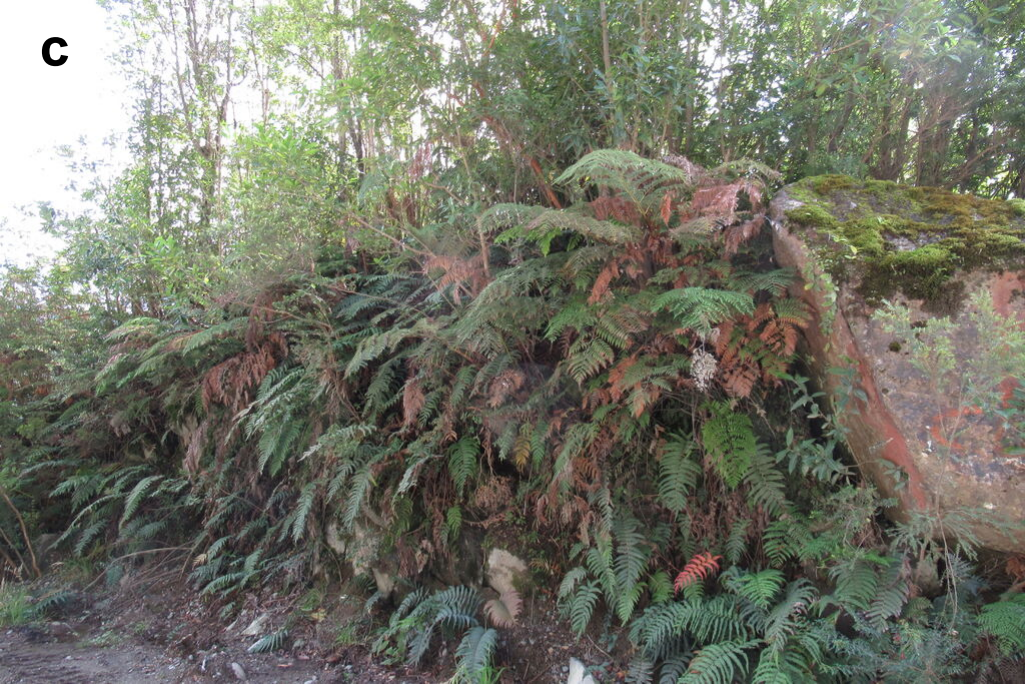
Isolated rock;

**Figure 2d.**
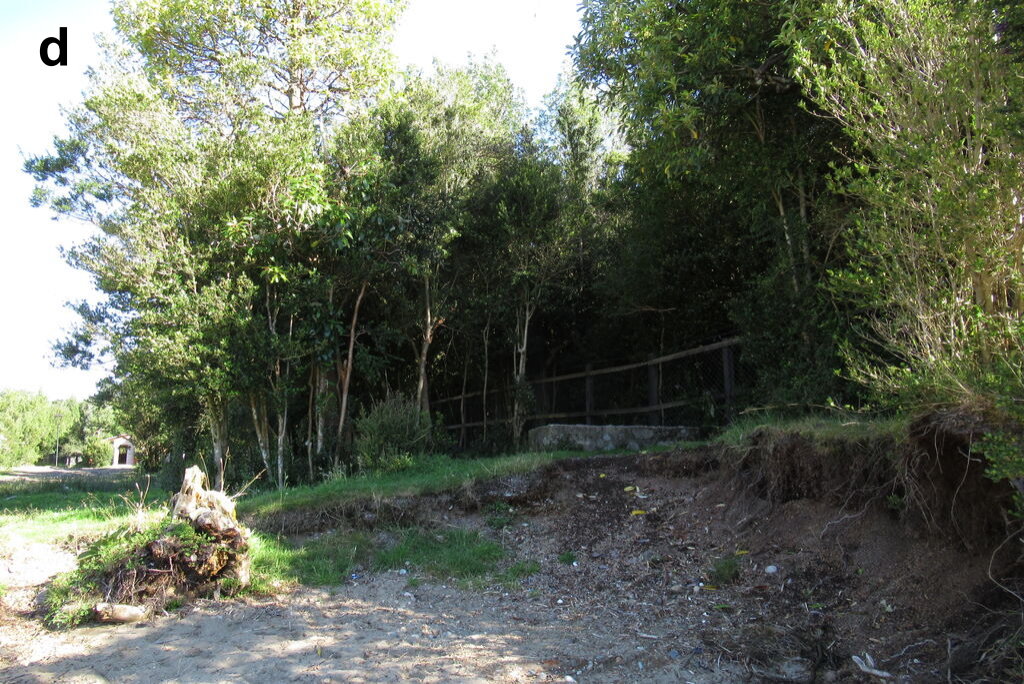
Transect 3.

**Figure 3a.**
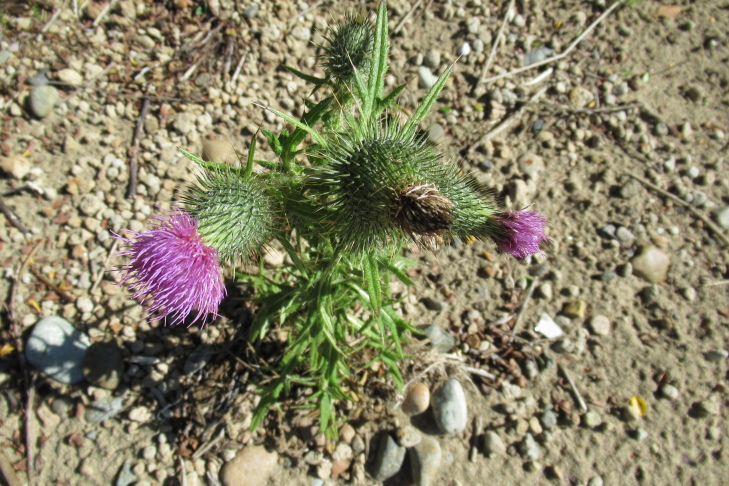
*Cirsiumvulgare*;

**Figure 3b.**
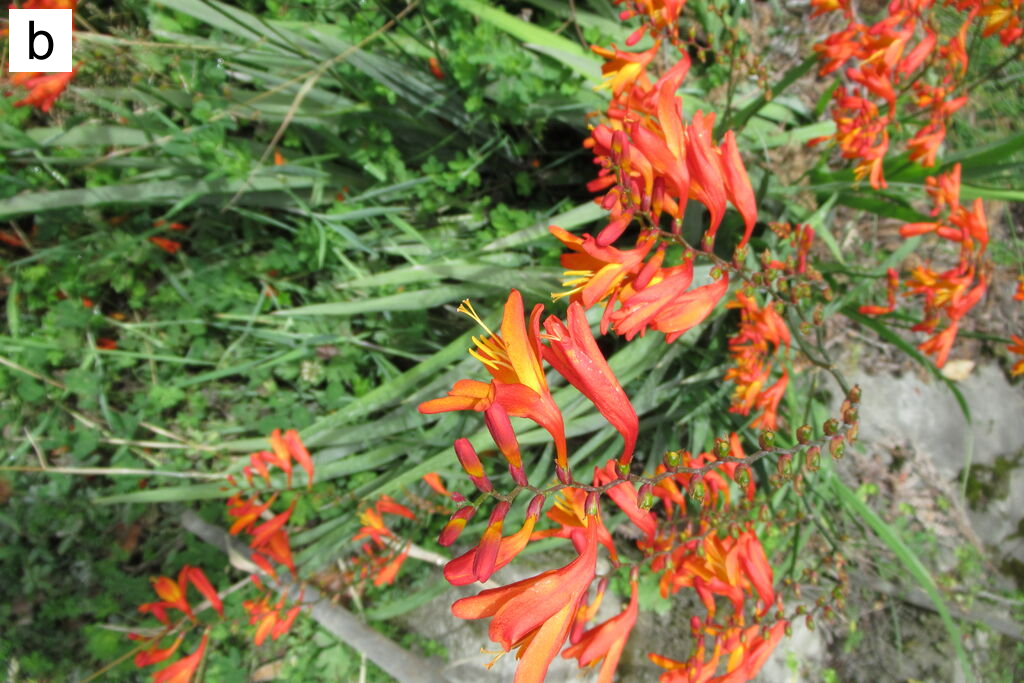
*Crocosmiacrocosmiiflora*;

**Figure 3c.**
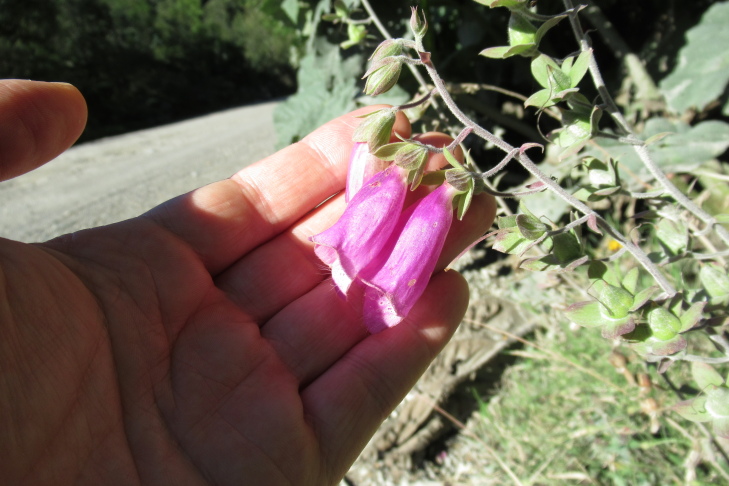
*Digitalispurpurea*;

**Figure 3d.**
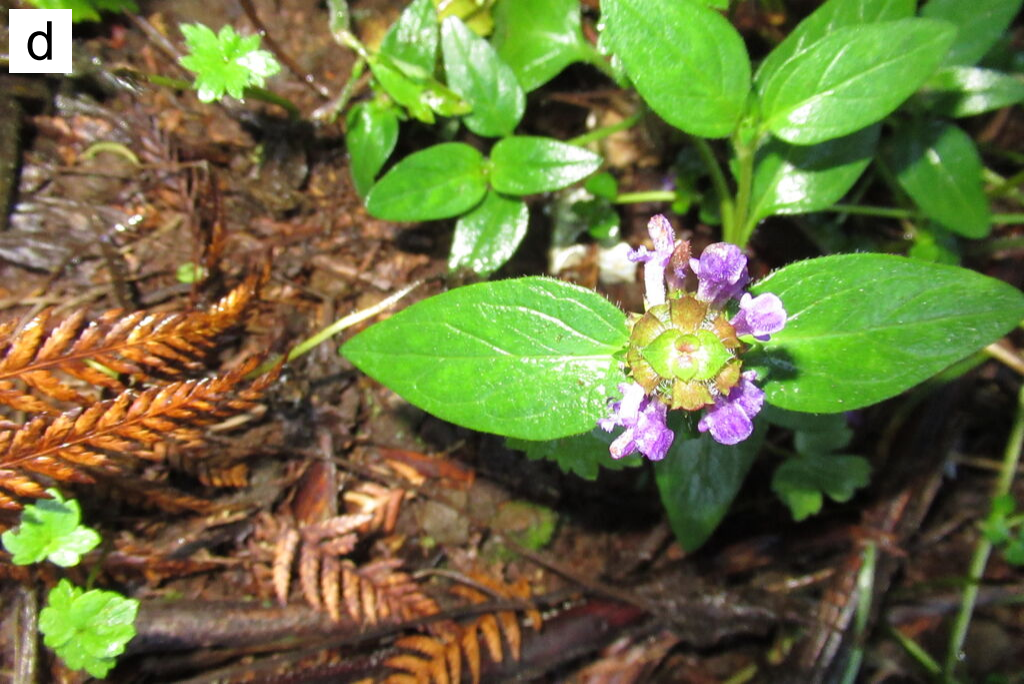
*Prunellavulgaris*;

**Figure 3e.**
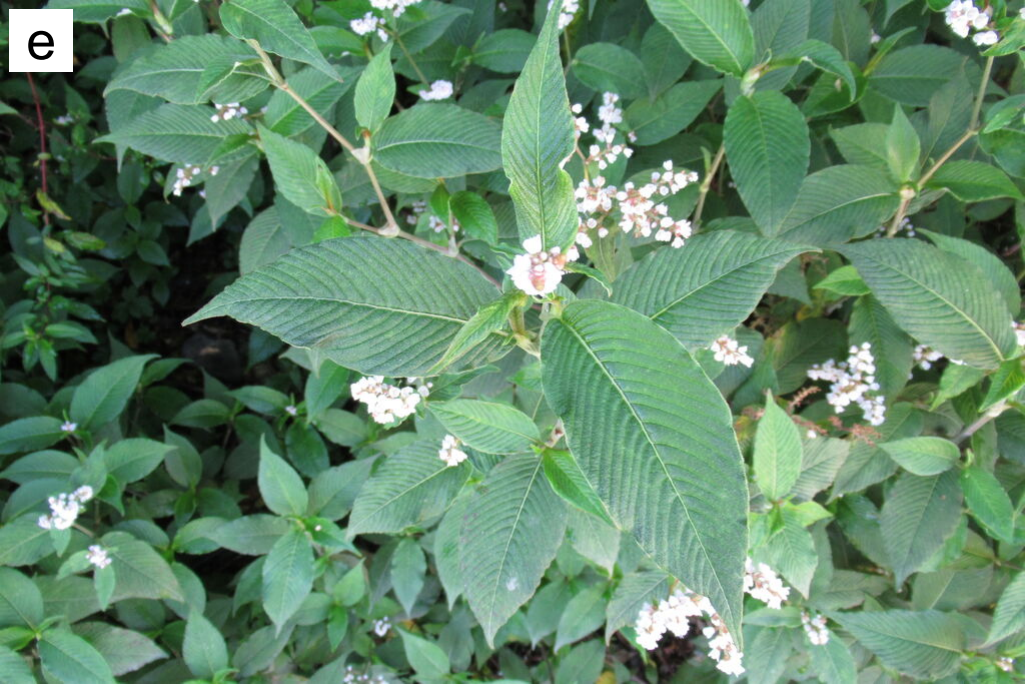
*Polygonumcampanulatum*;

**Figure 3f.**
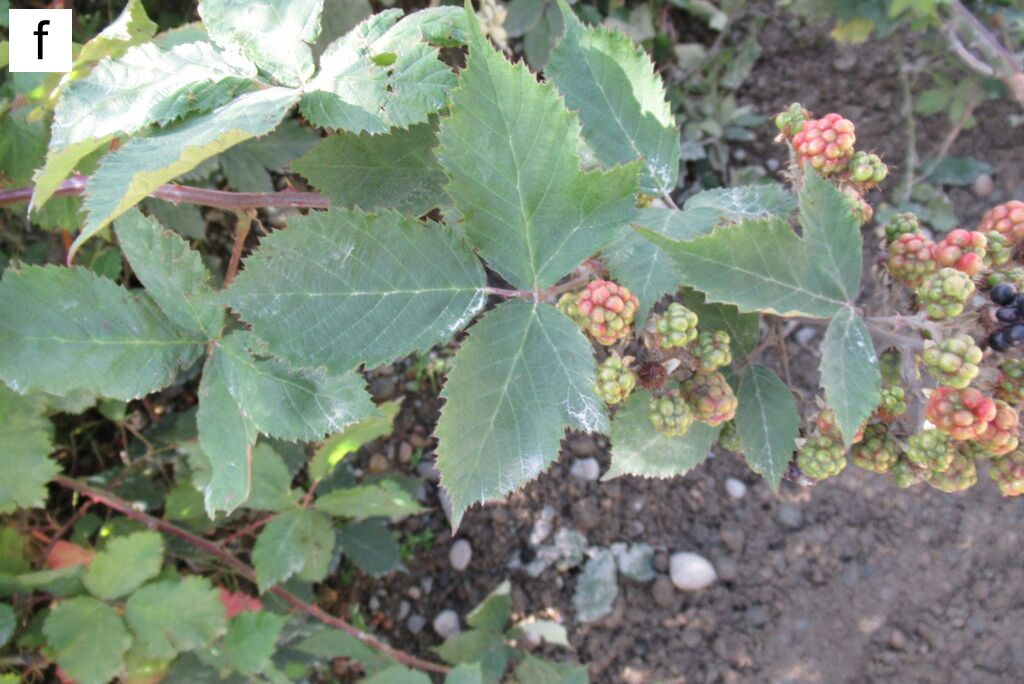
*Rubusconstrictus*.

**Figure 4a.**
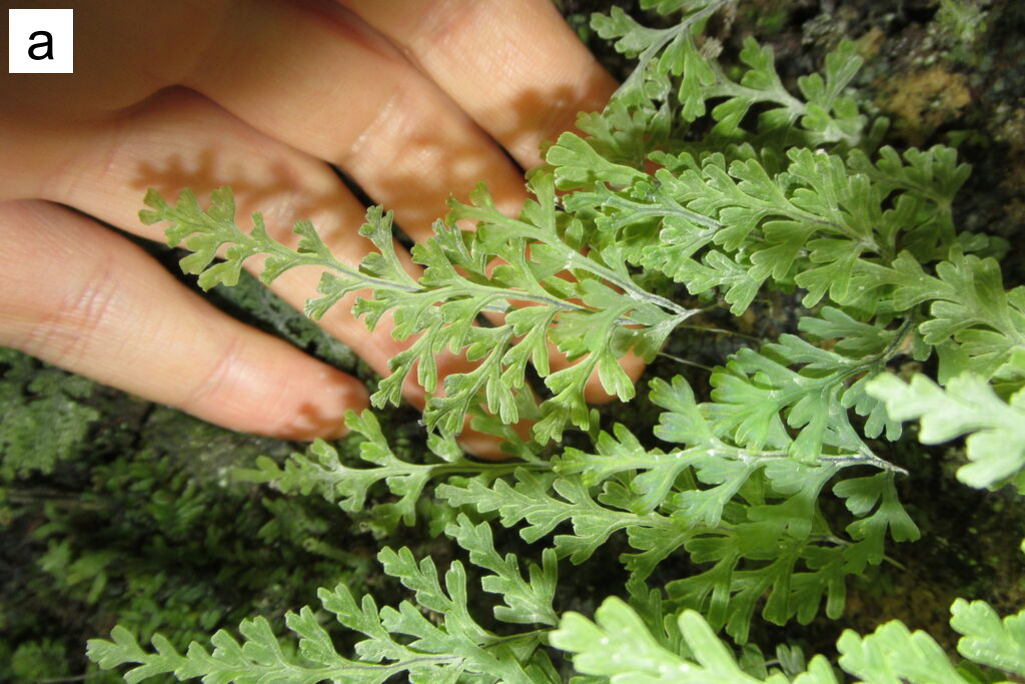
*Hymenophyllumcuneatum*;

**Figure 4b.**
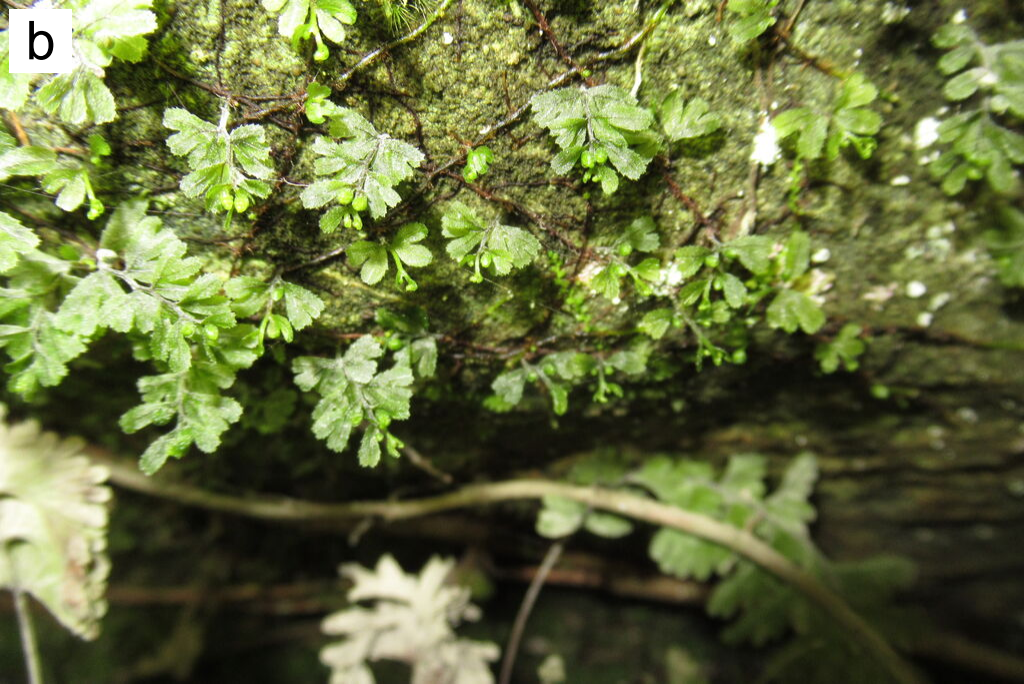
*Hymenophyllumfalklandicum*;

**Figure 4c.**
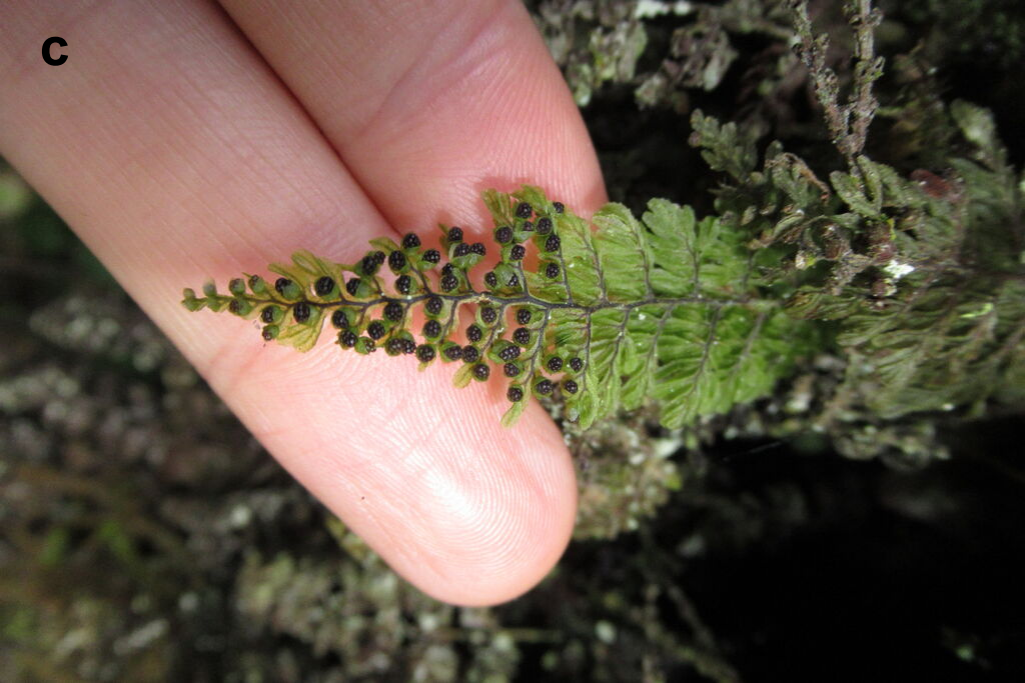
*Hymenophyllumdentatum*;

**Figure 4d.**
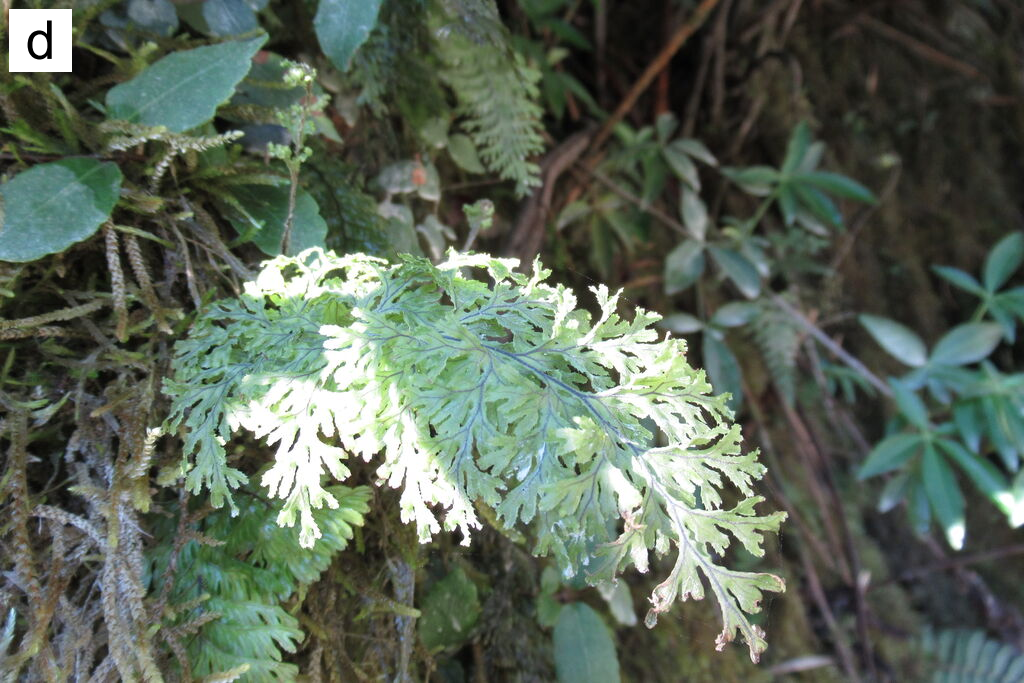
*Hymenophyllumkrauseanum*;

**Figure 4e.**
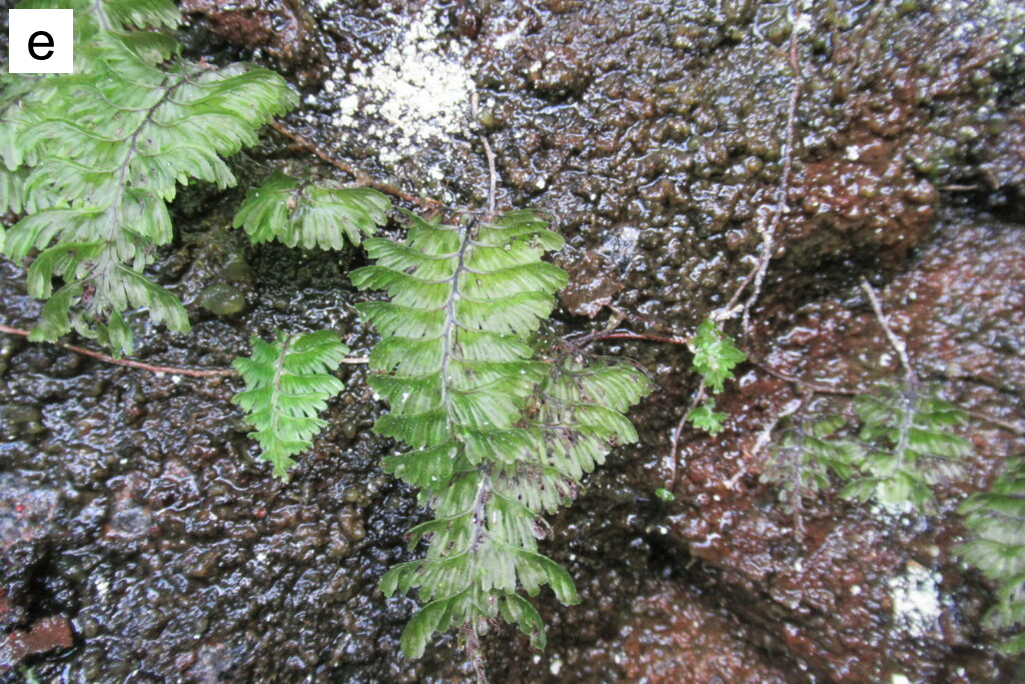
*Hymenophyllumpectinatum*;

**Figure 4f.**
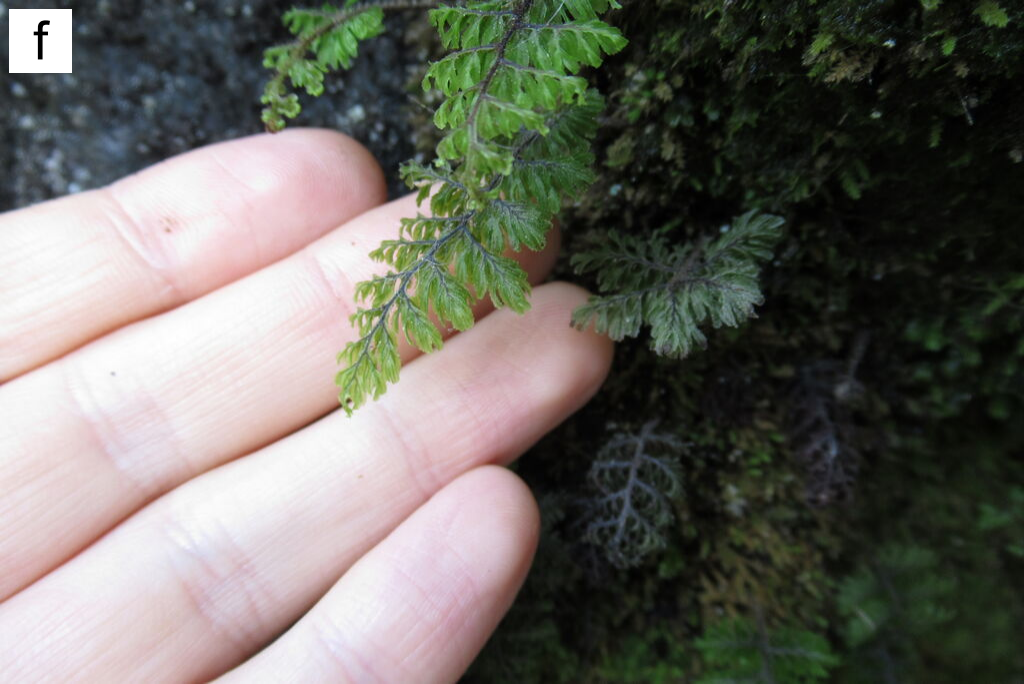
*Hymenophyllumplicatum*.

**Figure 5a.**
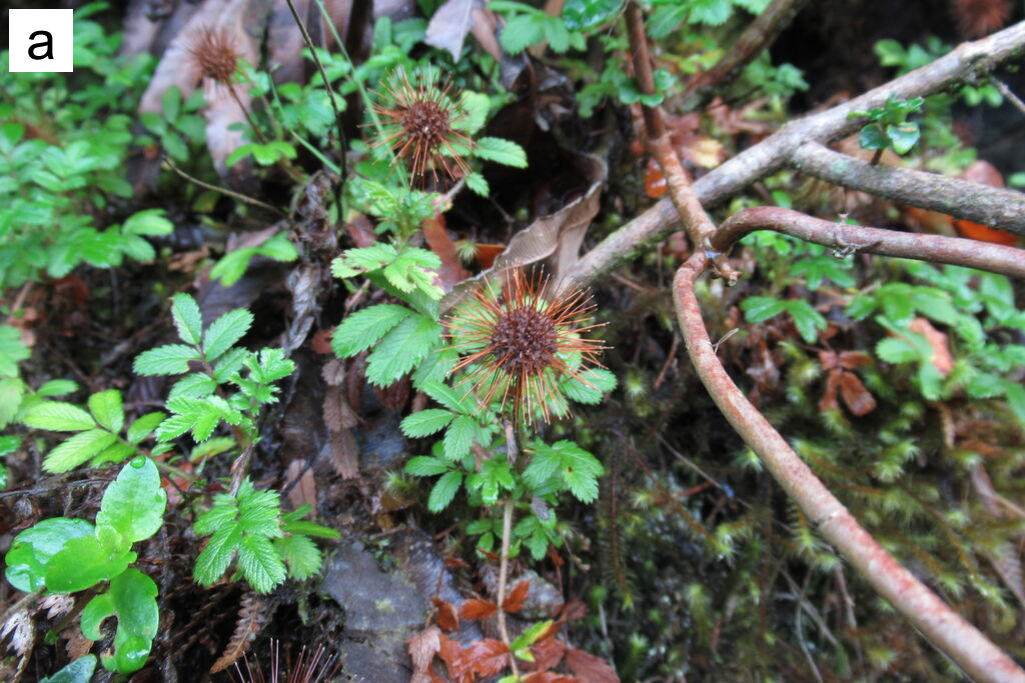
*Acaenaovalifolia*;

**Figure 5b.**
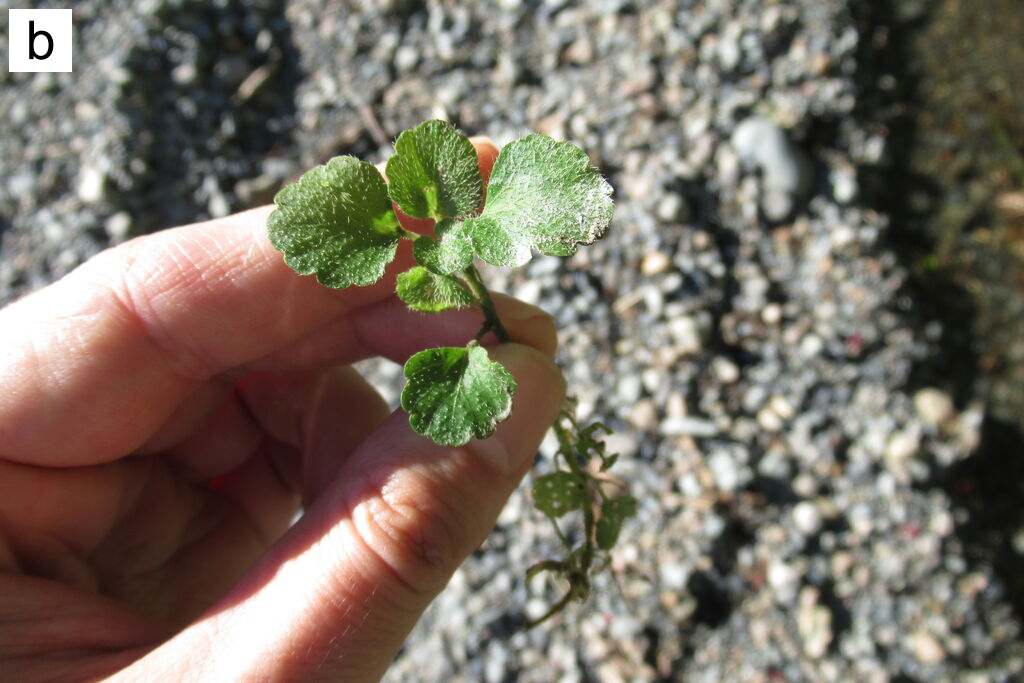
*Dysopsisglechomoides*;

**Figure 5c.**
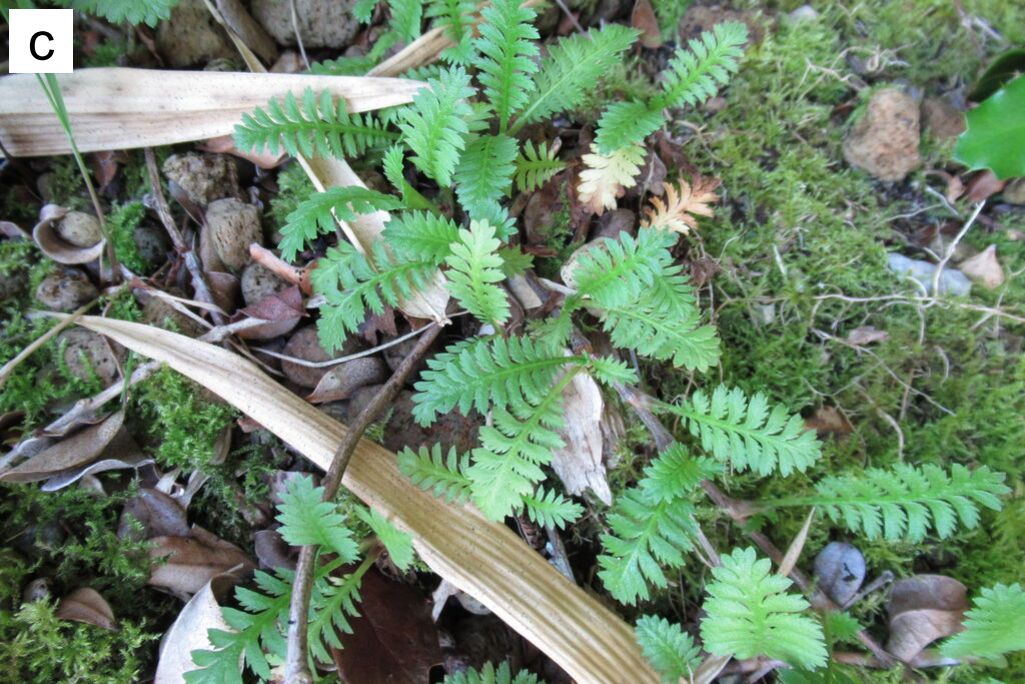
*Leptinellascariosa*;

**Figure 5d.**
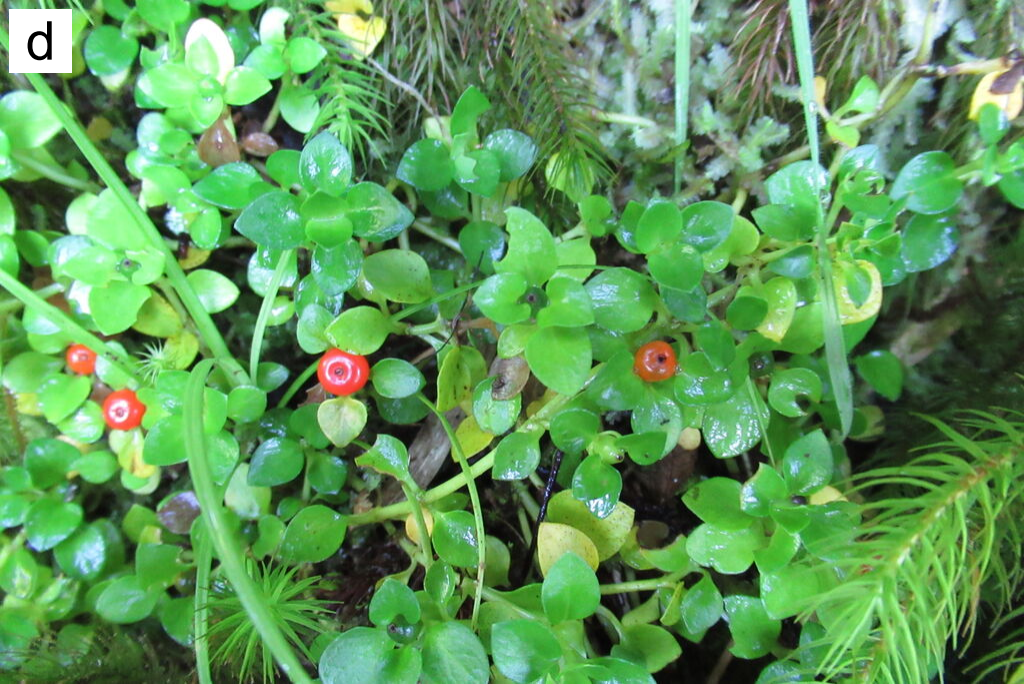
*Nerteragranadensis*;

**Figure 5e.**
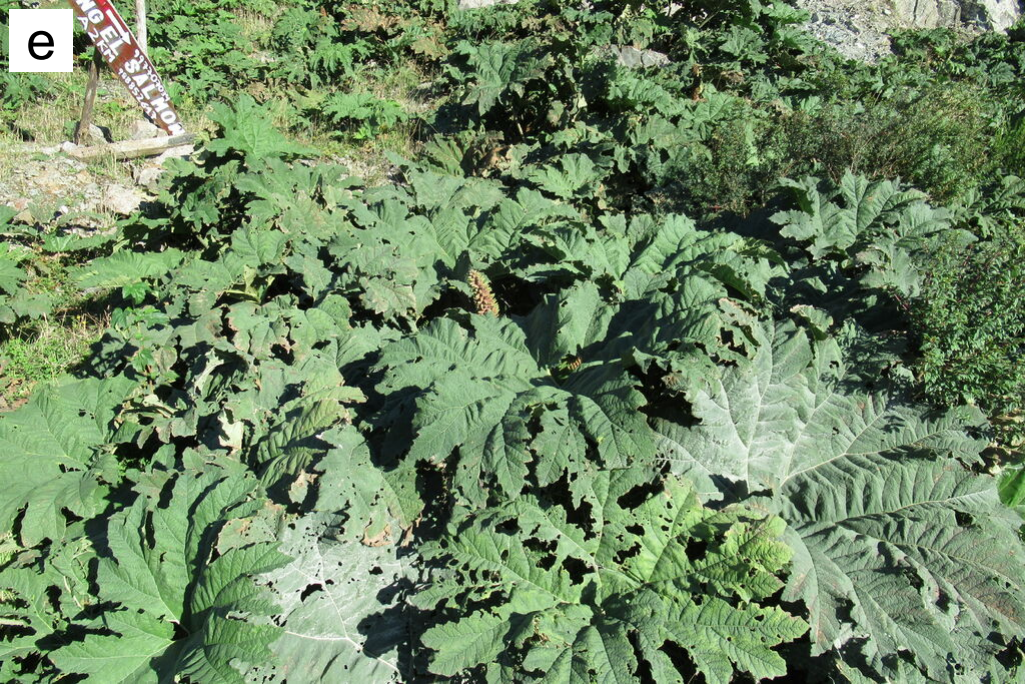
*Gunneratinctoria*;

**Figure 5f.**
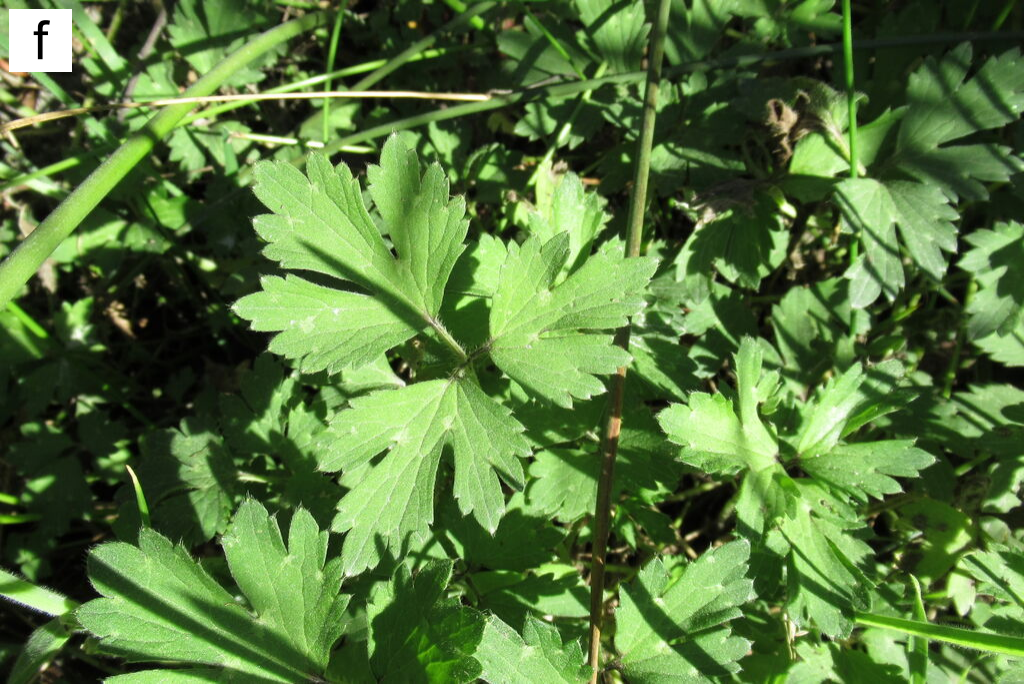
*Ranunculusrepens*.

**Figure 6a.**
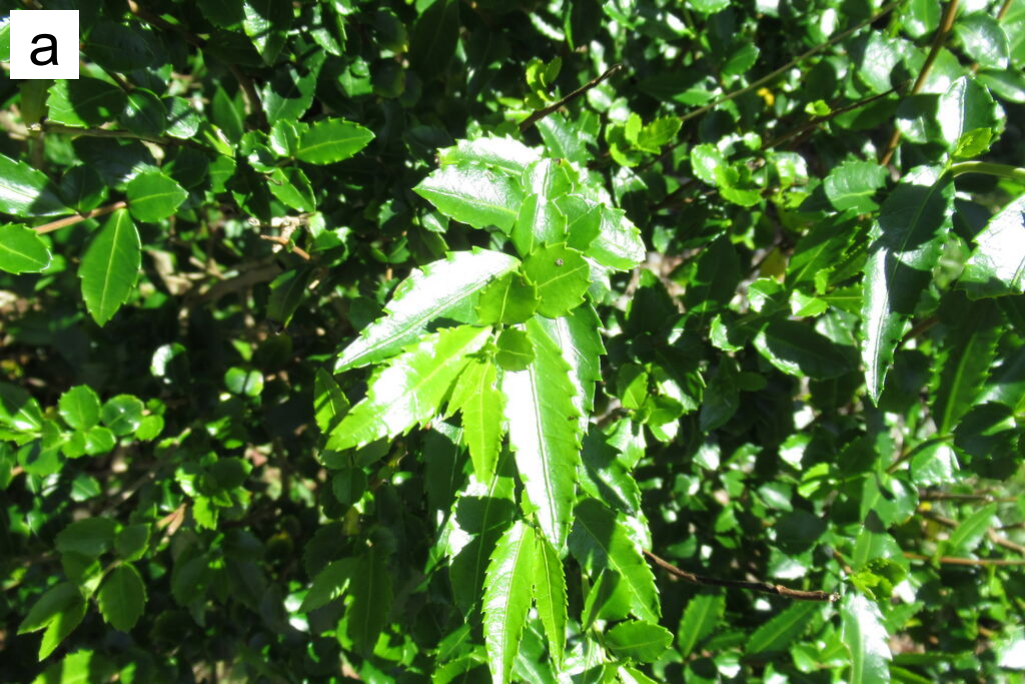
*Azaralanceolata*;

**Figure 6b.**
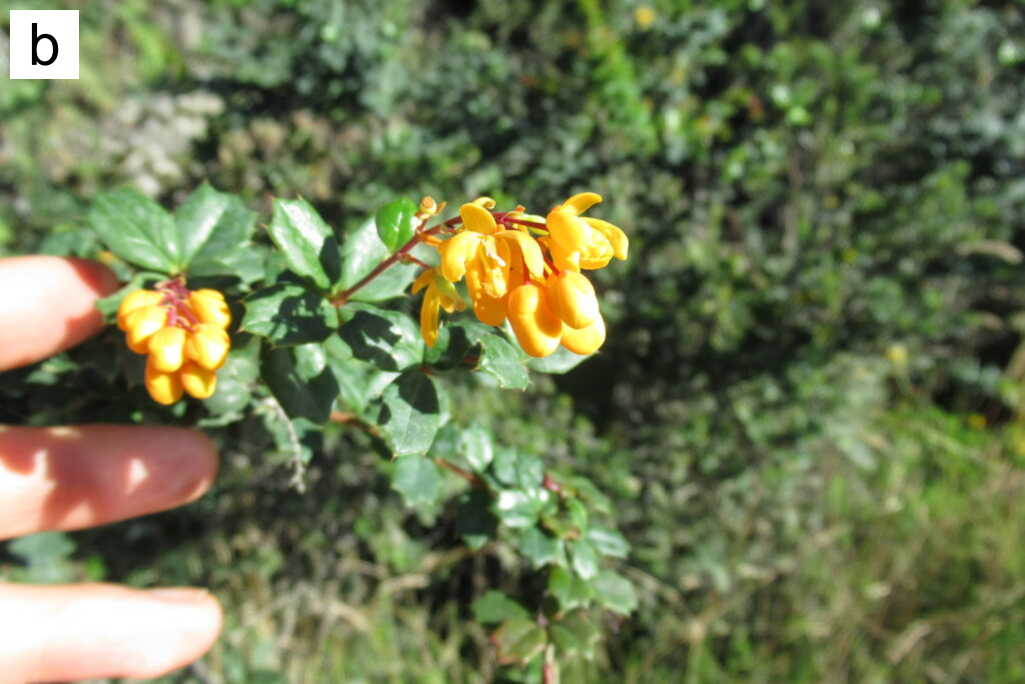
*Berberisdarwinii*;

**Figure 6c.**
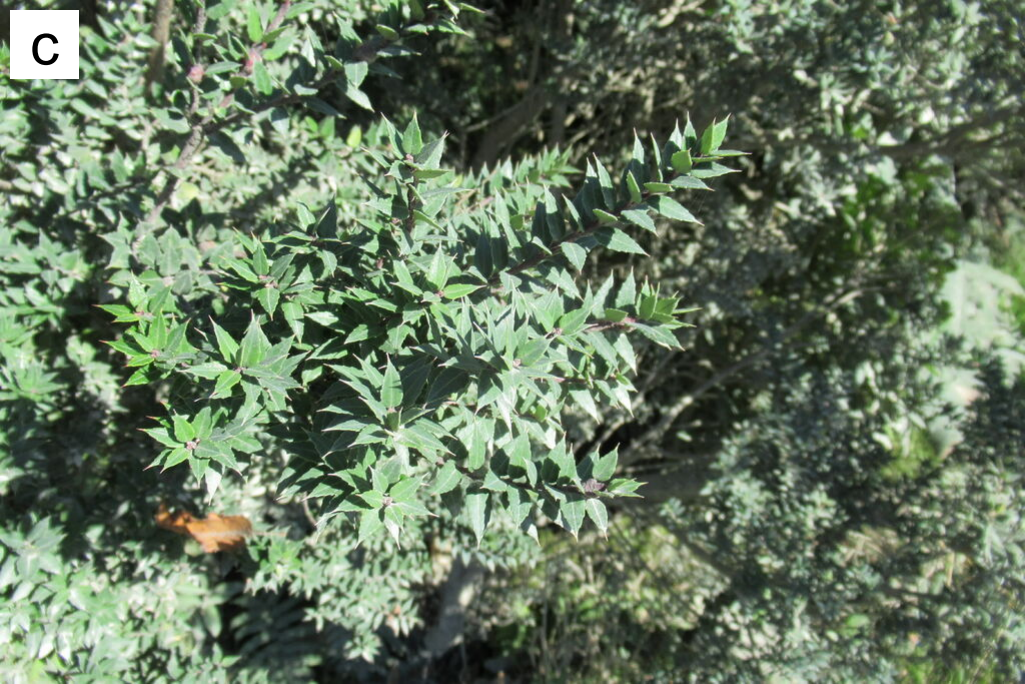
*Gaultheriaphillyreifolia*;

**Figure 6d.**
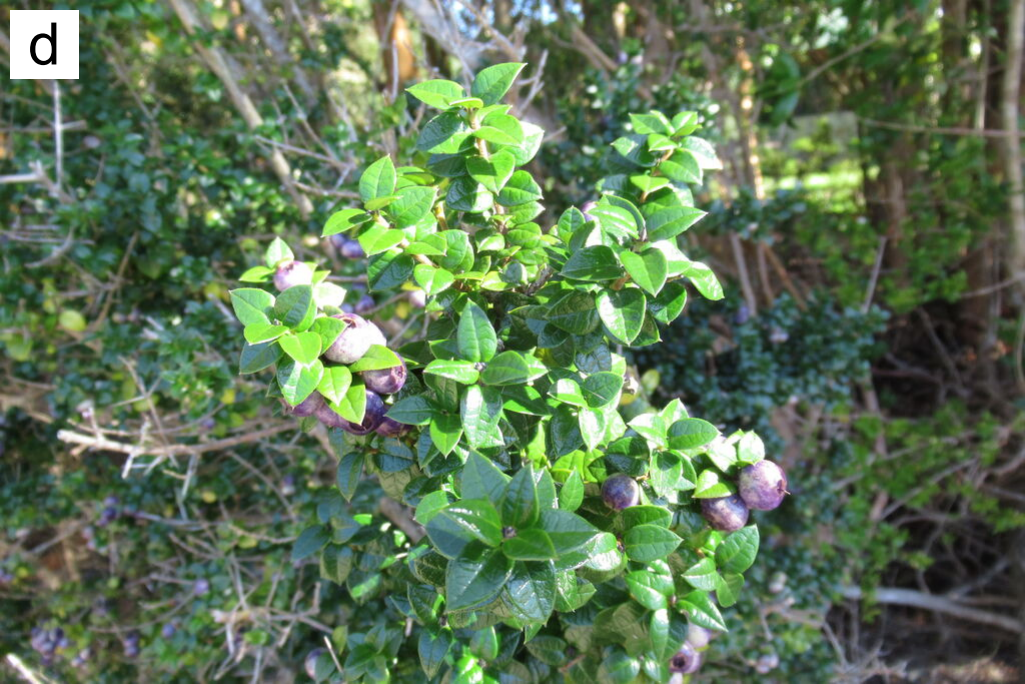
*Raphithamnusspinosus*;

**Figure 6e.**
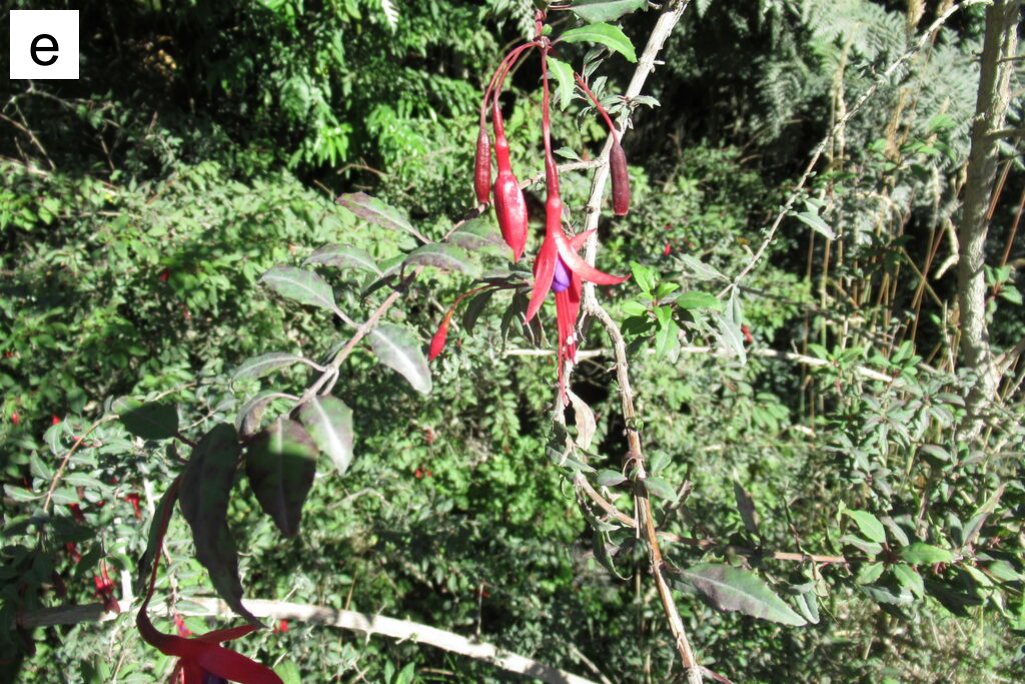
*Fuchsiamagellanica*;

**Figure 6f.**
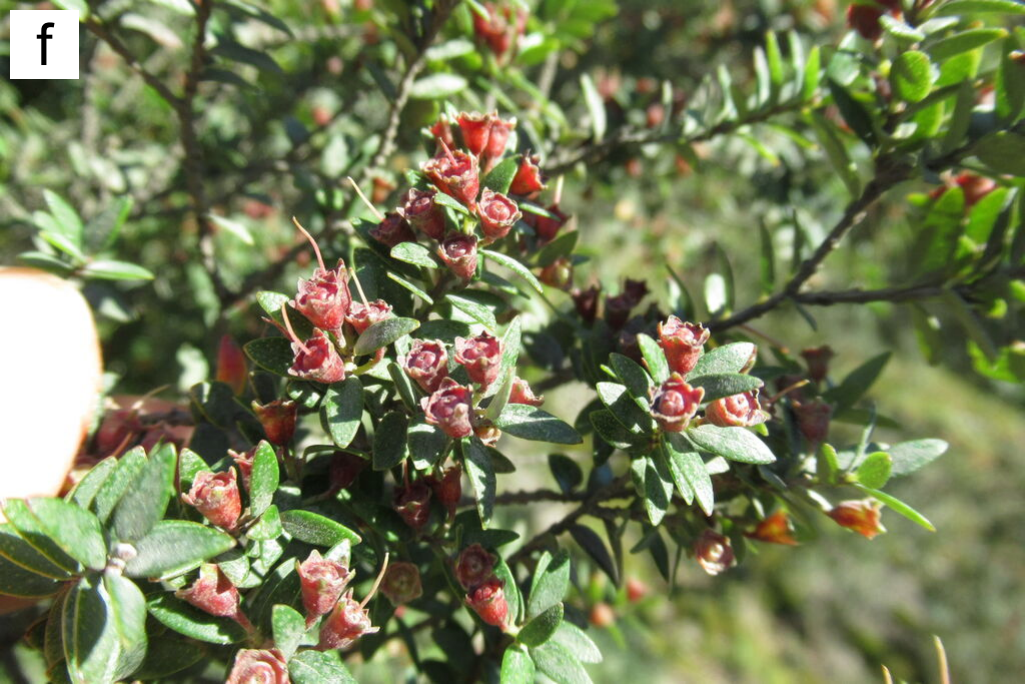
*Tepualiastipularis*.

**Figure 7a.**
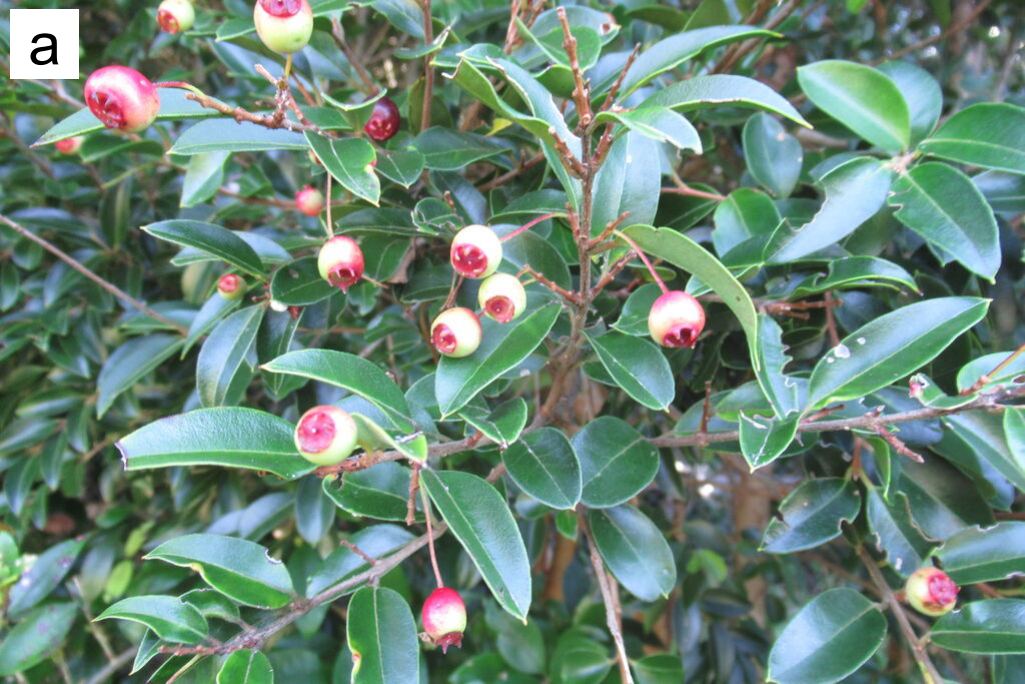
*Amomyrtusluma*;

**Figure 7b.**
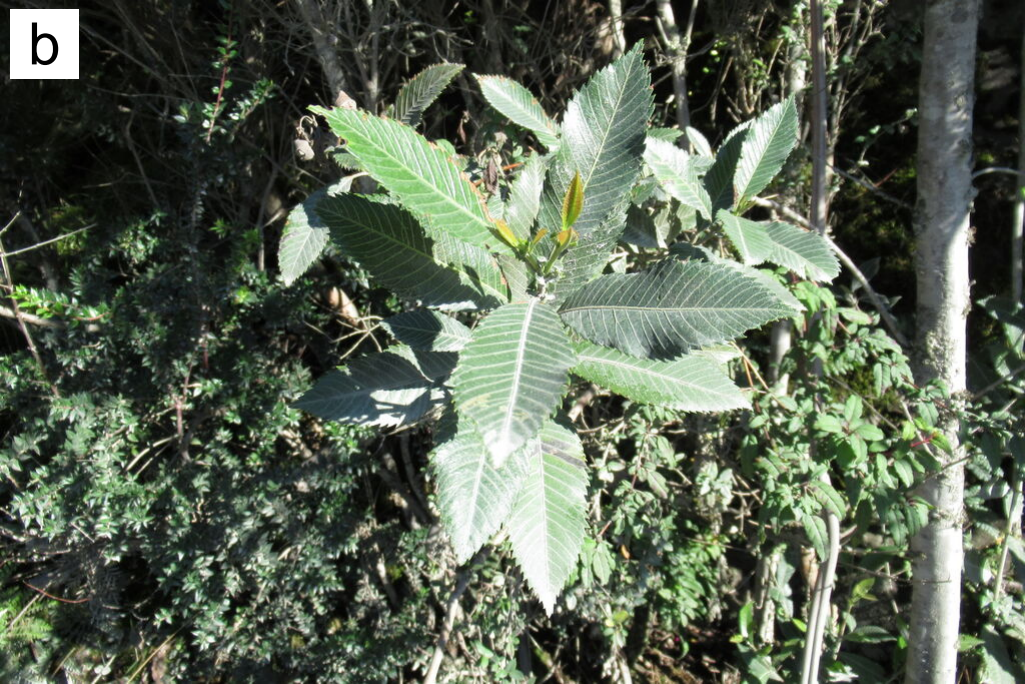
*Caldcluviapaniculata*;

**Figure 7c.**
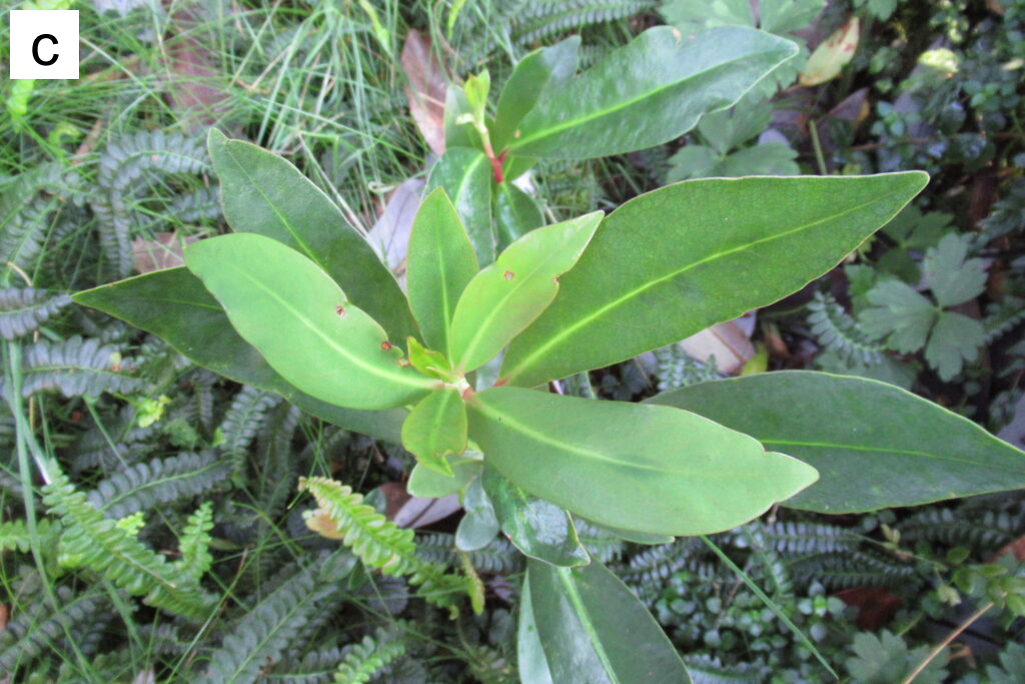
*Drimyswinteri*;

**Figure 7d.**
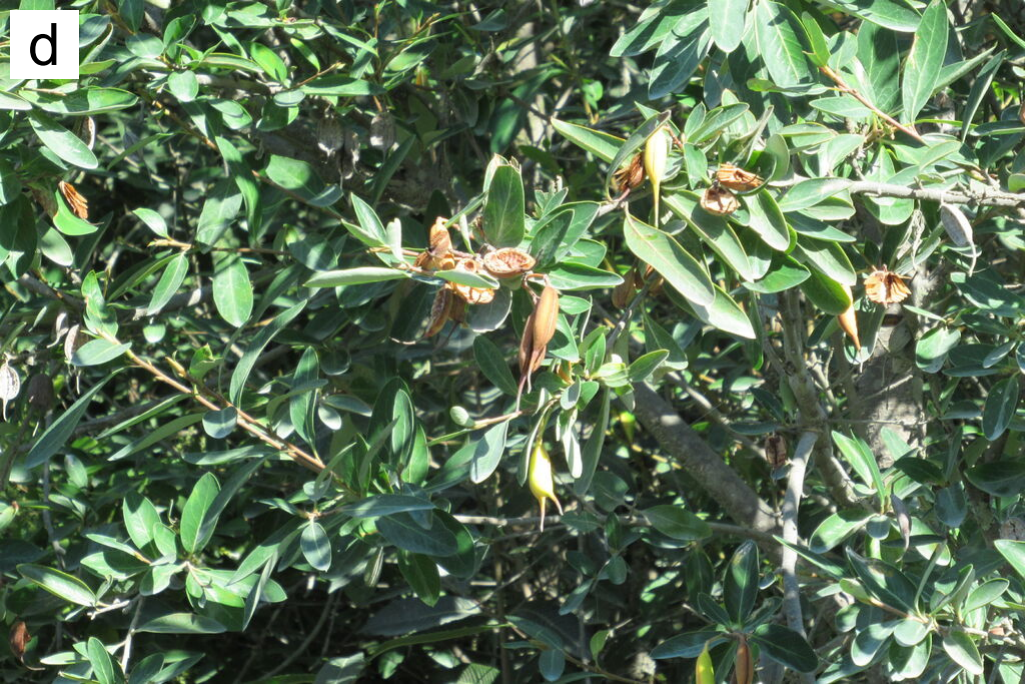
*Embothriumcoccineum*;

**Figure 7e.**
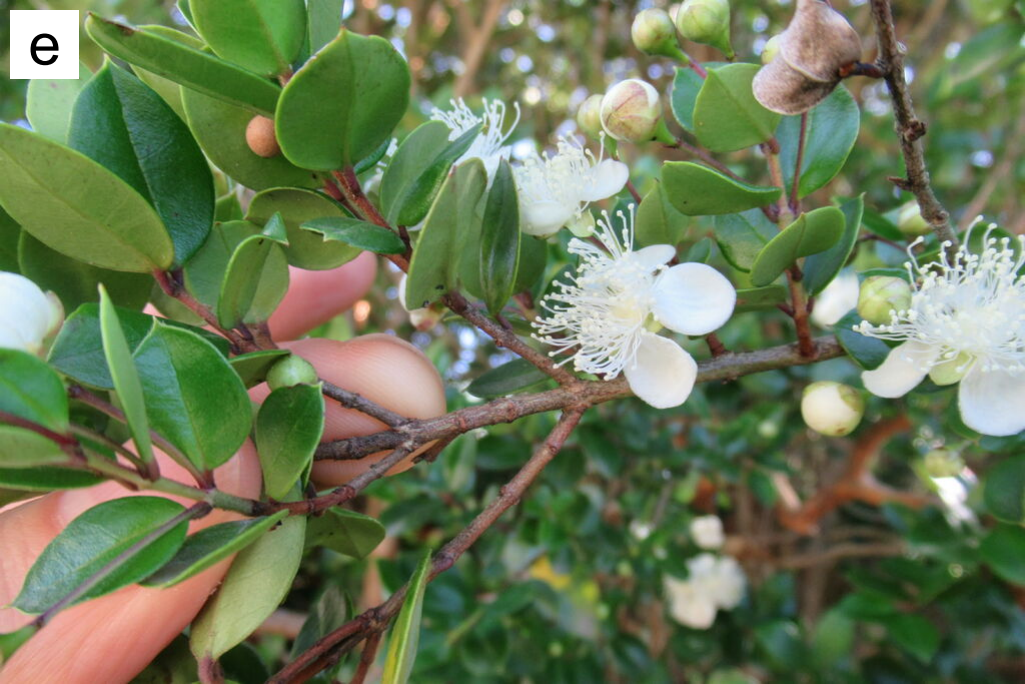
*Lumaapiculata*;

**Figure 7f.**
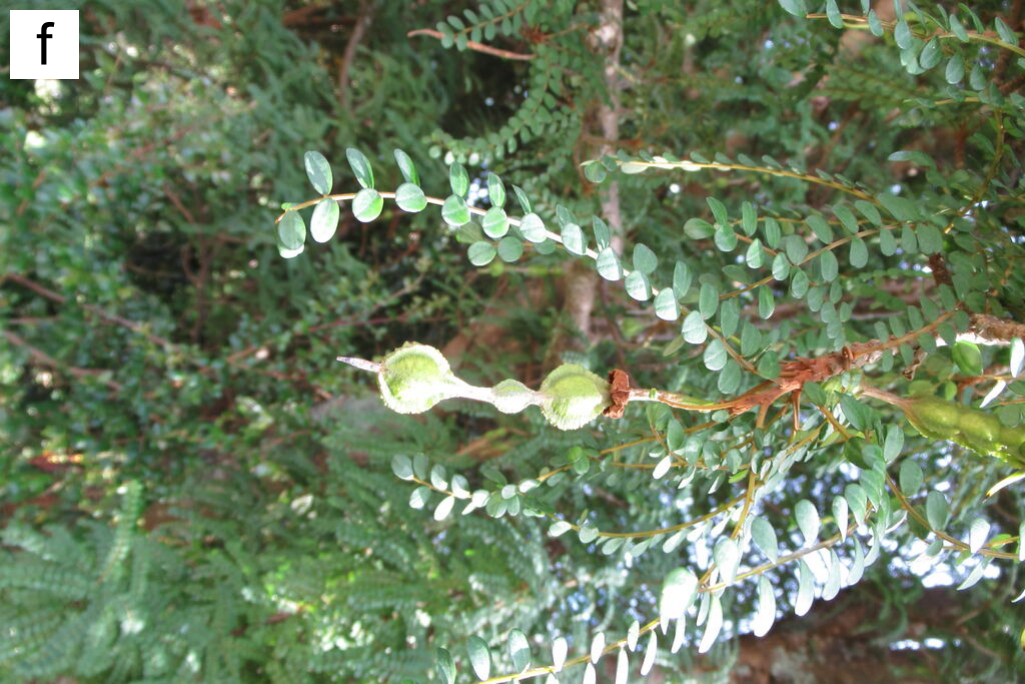
*Sophoracassioides*.

**Table 1.** Vascular plants along a coastal road in Puerto Cisnes, Aysén Region, Chile. Species: Scientific name of a species. Habit: Climbing shrub, herb, liana, shrub and tree. Site where species were observed: Transect 1, Transect 2, Isolated rock, Transect 3. Seed dispersal syndrome: anemochorous, ornithochorous, mammalochory, ballochory, myrmecochory according to Armesto and Rozzi (1989), Wilson et al. (1996) and Salvande et al. (2011). Geographical origin: native, endemic and introduced according to Rodriguez et al. (2018). *: Invasive species according to Fuentes et al. (2013) and Fuentes et al. (2020). 1: presence, 0: absence

Species	Habit	Transect 1	Transect 2	Isolated rock	Transect 3	Seed dispersal syndrome	Geographic origin
*Acaenaovalifolia* Ruiz & Pav.	Herb	1	1	0	0	Epizoochory	Native
*Acrisionecymosa* (J. Remy) B. Nord.	Shrub	0	1	0	0	Anemochory	Endemic
*Adiantumchilense* Kaulf.	Herb	1	1	0	0	Anemochory	Native
*Amomyrtusluma* (Molina) D. Legrand & Kausel	Tree	1	1	0	1	Ornithochory	Native
*Aristoteliachilensis* (Molina) Stuntz	Tree	0	1	0	0	Ornithochory	Native
*Aspleniumdareoides* Desv.	Herb	1	0	0	1	Anemochory	Native
*Aspleniumtrilobum* Cav.	Herb	0	1	1	1	Anemochory	Native
*Asterantheraovata* (Cav.) Hanst.	Shrub	1	1	0	0	Ornithochory	Native
*Azaralanceolata* Hook.f.	Shrub	1	1	0	1	Ornithochory	Native
*Berberisdarwinii* Hook.	Shrub	1	1	0	0	Ornithochory	Native
*Berberismicrophylla* G. Forst.	Shrub	0	1	0	0	Ornithochory	Native
*Blechnumchilense* (Kaulf.) Mett.	Herb	1	1	1	0	Anemochory	Native
*Blechnumpenna-marina* (Poir.) Kuhn	Herb	0	1	0	1	Anemochory	Native
*Caldcluviapaniculata* (Cav.) D. Don	Tree	1	1	1	0	Anemochory	Native
*Campsidiumvaldivianum* (Phil.) Skottsb.	Shrub	1	1	0	0	Anemochory	Native
*Diplolepispachyphylla* (Decne.) Hechem & C. Ezcurra	Herb	0	0	0	1	Anemochory	Native
*Chusqueaculeou* E. Desv.	Herb	0	0	0	1	Anemochory	Native
*Cirsiumvulgare* (Savi) Ten.	Herb	0	0	0	1	Anemochory	Introduced*
*Crocosmiacrocosmiiflora* (Lemoine ex Burb. & Dean) N.E.Br.	Herb	0	1	0	0	Hydrochory/zoochory	Introduced
*Cytisusscoparius* (L.) Link	Shrub	0	1	0	0	Ballochory/ myrmecochory	Introduced*
*Digitalispurpurea* L.	Herb	0	1	0	0	Multiple	Introduced*
*Drimyswinteri* J.R. Forst. & G. Forst.	Tree	1	0	0	1	Ornithochory	Endemic
*Dysopsisglechomoides* (A. Rich.) Müll. Arg.	Herb	0	1	0	0	Ballochory/ myrmecochory	Endemic
*Embothriumcoccineum* J.R. Forst. & G. Forst.	Tree	0	1	0	0	Anemochory	Native
*Ercillasyncarpellata* Nowicke	Shrub	0	1	0	0	Ornithochory	Endemic
*Fasciculariabicolor* (Ruiz & Pav.) Mez	Herb	0	1	0	0	Ornithochory	Endemic
*Fuchsiamagellanica* Lam.	Shrub	1	1	0	0	Ornithochory	Native
*Galiumhypocarpium* (L.) Endl. ex Griseb.	Herb	1	1	0	1	Mammalochory/saurochory	Native
*Gaultheriaphillyreifolia* (Pers.) Sleumer	Shrub	0	1	0	0	Ornithochory/saurochory	Native
*Griseliniaracemosa* (Phil.) Taub.	Shrub	1	1	1	0	Ornithochory	Native
*Gunneramagellanica* Lam.	Herb	0	1	0	0	Mammalochory	Native
*Gunneratinctoria* (Molina) Mirb.	Herb	0	1	0	0	Mammalochory	Native
*Hydrangeaserratifolia* (Hook. & Arn.) F. Phil.	Shrub	1	1	0	0	Multiple	Native
*Hymenoglossumcruentum* (Cav.) C. Presl	Herb	1	0	0	0	Anemochory	Native
*Hymenophyllumcuneatum* Kunze	Herb	1	0	1	0	Anemochory	Endemic
*Hymenophyllumdentatum* Cav.	Herb	1	1	1	1	Anemochory	Native
*Hymenophyllumfalklandicum* Baker	Herb	0	0	1	0	Anemochory	Native
*Hymenophyllumkrauseanum* Phil.	Herb	1	1	0	0	Anemochory	Native
*Hymenophyllumpectinatum* Cav.	Herb	1	1	0	0	Anemochory	Native
*Hymenophyllumpeltatum* (Poir.) Desv.	Herb	1	0	1	1	Anemochory	Native
*Hymenophyllumplicatum* Kaulf.	Herb	1	1	1	0	Anemochory	Native
*Laureliopsisphilippiana* (Looser) Schodde	Tree	1	1	1	1	Anemochory	Native
*Leptinellascariosa* Cass.	Herb	0	1	0	0	Anemochory	Native
*Lomatiaferruginea* (Cav.) R. Br.	Tree	1	1	0	0	Anemochory	Native
*Lophosoriaquadripinnata* (J.F. Gmel.) C. Chr.	Herb	1	1	1	0	Anemochory	Native
*Lotuspedunculatus* Cav.	Herb	0	1	0	0	Anemochory	Introduced*
*Lumaapiculata* (DC.) Burret	Tree	0	1	0	1	Ornithochory	Native
*Luzuriagapolyphylla* (Hook.) J.F. Macbr.	Subshrub	1	1	0	1	Ornithochory	Endemic
*Luzuriagaradicans* Ruiz & Pav.	Subshrub	0	1	0	0	Ornithochory	Native
*Megalastrumspectabile* (Kaulf.) A.R. Sm. & R.C. Moran	Herb	0	1	0	0	Anemochory	Native
*Mitrariacoccinea* Cav.	Shrub	1	1	0	0	Ornithochory	Native
*Myrceugeniaplanipes* (Hook. & Arn.) O. Berg	Tree	0	0	0	1	Ornithochory	Native
*Nerteragranadensis* (Mutis ex L.f.) Druce	Herb	1	1	0	0	Ornithochory/saurochory	Native
*Philesiamagellanica* J.F. Gmel.	Subshrub	0	1	0	0	Ornithochory	Native
*Plantagoaustralis* Lam.	Herb	0	1	0	0	Hydrochory	Endemic
*Plantagolanceolata* L.	Herb	0	1	0	0	Hydrochory	Introduced*
*Polygonumcampanulatum* Hook. f.	Herb	1	0	0	0	Hydrochory/zoochory	Introduced
*Prunellavulgaris* L.	Herb	0	1	0	0	Myrmecochory	Introduced*
*Ranunculusrepens* L.	Herb	0	1	0	0	Hydrochory	Native
*Rhaphithamnusspinosus* (Juss.) Moldenke	Shrub	0	0	0	1	Ornithochory	Native
*Raukaualaetevirens* (Gay) Frodin	Shrub	0	1	0	1	Ornithochory	Native
*Ribesmagellanicum* Poir.	Shrub	1	1	0	0	Ornithochory	Native
*Rubusconstrictus* Lefèvre & P.J.Müll	Shrub	0	1	0	0	Endozoochory	Introduced*
*Sarmientascandens* (J.D. Brandis ex Molina) Pers.	Shrub	0	1	0	0	Anemochory	Endemic
*Serpyllopsiscaespitosa* (Gaudich.) C. Chr.	Herb	1	0	1	1	Anemochory	Native
*Sophoracassioides* (Phil.) Sparre	Tree	0	0	0	1	Hydrochory	Endemic
*Sticherussquamulosus* (Desv.) Nakai	Herb	0	1	0	0	Anemochory	Endemic
*Synammiafeuillei* (Bertero) Copel.	Herb	0	0	0	1	Anemochory	Native
*Tepualiastipularis* (Hook. & Arn.) Griseb.	Shrub	1	1	0	0	Anemochory	Native
*Weinmanniatrichosperma* Cav.	Tree	0	1	0	0	Anemochory	Native
